# Induction of miR-3648 Upon ER Stress and Its Regulatory Role in Cell Proliferation

**DOI:** 10.3390/ijms18071375

**Published:** 2017-06-29

**Authors:** Farooq Rashid, Hassaan Mehboob Awan, Abdullah Shah, Liang Chen, Ge Shan

**Affiliations:** Chinese Academy of Sciences (CAS) Key Laboratory of Innate Immunity and Chronic Disease, CAS Center for Excellence in Molecular Cell Science, School of Life Sciences, University of Science and Technology of China, Hefei 230027, China; farooq12@mail.ustc.edu.cn (F.R.); hassaan@mail.ustc.edu.cn (H.M.A.); shah@mail.ustc.edu.cn (A.S.); anqingcl@ustc.edu.cn (L.C.)

**Keywords:** miR-3648, endoplasmic reticulum stress, Adenomatous polyposis coli 2, proliferation, Wnt

## Abstract

MicroRNAs (miRNAs) play important roles under multiple cellular conditions including endoplasmic reticulum (ER) stress. We found that miR-3648, a human specific microRNA, was induced under ER stress. Moreover, Adenomatous polyposis coli 2 (APC2), a tumor suppressor and a negative regulator of Wnt signaling, was found to be the direct target of miR-3648. Levels of APC2 were downregulated when cells were under ER stress or after overexpressing miR-3648. Inhibition of miR-3648 by antagomir increased APC2 levels and decreased cell proliferation. Conversely, when miR-3648 was overexpressed, APC2 levels were decreased and the cell growth increased. Our data demonstrated that ER stress mediated induction of miR-3648 in human cells, which then downregulated APC2 to increase cell proliferation.

## 1. Introduction

MicroRNAs (miRNAs) are small ~22-nucleotides noncoding RNAs (ncRNAs). Generally, miRNAs bind to 3′ untranslated region (3′ UTR) of target messenger RNAs (mRNAs) to promote mRNA degradation or repress translation [1,2]. miRNA gene is transcribed to give rise to a large primary transcript (primary microRNA, Pri-miR), which is then processed sequentially to produce the mature miRNA [3,4]. miRNAs are involved in many biological processes including tumorigenesis, and participate in response to various stresses such as oxidative stress and endoplasmic reticulum (ER) stress [5,6,7,8,9].

ER is responsible for folding and maturation of a large number of proteins [10,11]. Protein folding is both regulated and sensed by ER resident chaperones, such as GRP78/Bip and Grp94 [12,13,14]. ER stress is a perturbed state caused by unresolved folding of proteins, which accumulate within the ER and cause cell toxicity. Thapsigargin (TG) and Tunicamycin (TM) are two drugs often used to generate ER stress in experimental setup [15]. Cells respond to ER stress by activating a protective response called unfolded protein response (UPR) [10,16]. Mammalian UPR is controlled by inositol-requiring enzymes 1 (IRE1), protein kinase-like ER kinase (PERK), and the activating transcription factor 6 (ATF6) [11,17,18]. IRE1 trigger increased expression of various ER chaperones by activating the X box binding protein 1 (XBP1) transcription factor [19]. PERK is activated in a manner similar to IRE1. It catalyzes serine 51 phosphorylation of eIF2α, resulting in global repression of protein synthesis [20]. ATF6 is involved in the transcriptional induction of ER chaperones [21].

ER stress has very complex effects [17]. The UPR will alleviate the stress conditions and restore the homeostasis of the cell. Tumors can have adaptive ER stress response, which occurs mainly through the activation of GRP78, to enhance tumor cell survival, proliferation, metastasis, and even drug resistance [17,22]. Generally, when ER stress is prolonged, and the protein load on ER exceeds its folding capacity, cell death may occur through a series of complementary pathways [23]. ER stress alters an array of cell signaling cascades including the Wnt/β-catenin pathway [24,25]. Multiple miRNAs have also been shown to participate in cellular responses to ER stress [26].

Adenomatous polyposis coli 2 (APC2) is a tumor suppressor and is expressed in different tissues and cell lines [27]. This protein binds with β-catenin, and negatively regulates Wnt/β-catenin signaling pathway [28,29]. Thus, the reduced levels of APC2 lead to high expression of downstream genes of the Wnt/β-catenin signaling pathway, and result in tumorigenesis, as activated Wnt signaling promotes cell proliferation [27,29,30,31].

In this study, we identified miR-3648, a human specific miRNA, to be upregulated in cells under ER stress. The tumor suppressor APC2 was found to be the major target of miR-3648 in ER stressed cells. By targeting APC2, miR-3648 played critical roles in regulating cell proliferation under ER stressed condition.

## 2. Results

### 2.1. Expression of miR-3648 in Human Cell Lines and Tissues

**Figure 1:**
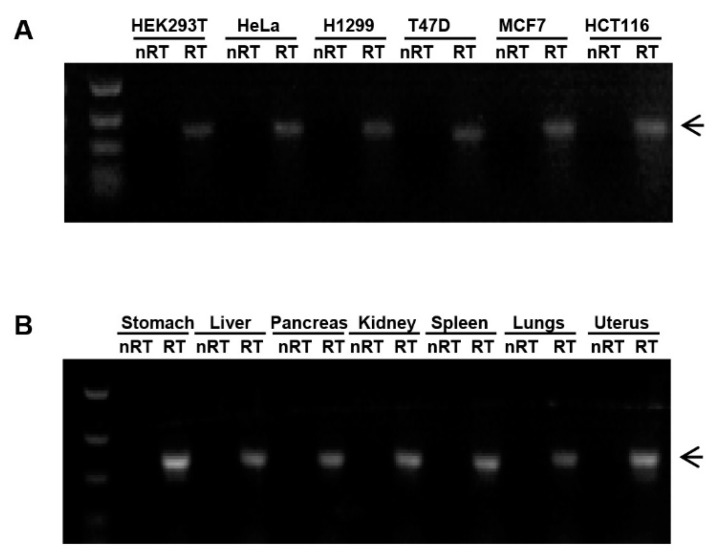
Expression of miR-3648 in human cell lines and tissues: (**A**) expression of miR-3648 in multiple cell lines by RT-PCR; and (**B**) expression of miR-3648 in multiple tissues by RT-PCR. miRNA bands were indicated with arrowheads. RT, Reverse transcription.

miR-3648 is a human specific miRNA, and no ortholog exists in great apes and the other mammals [32]. Sequencing data previously generated in our lab showed miR-3648 to be upregulated in ER stress [26]. We checked the endogenous expression of miR-3648 in different human cell lines and tissues, and found this miRNA to be expressed in all the tested cell lines and tissues (Figure 1A,B). These data indicated that miR-3648 might play physiological roles in many human cells, and its expression might be regulated in conditions such as ER stress.

### 2.2. miR-3648 Was Induced by the ER Stress

**Figure 2:**
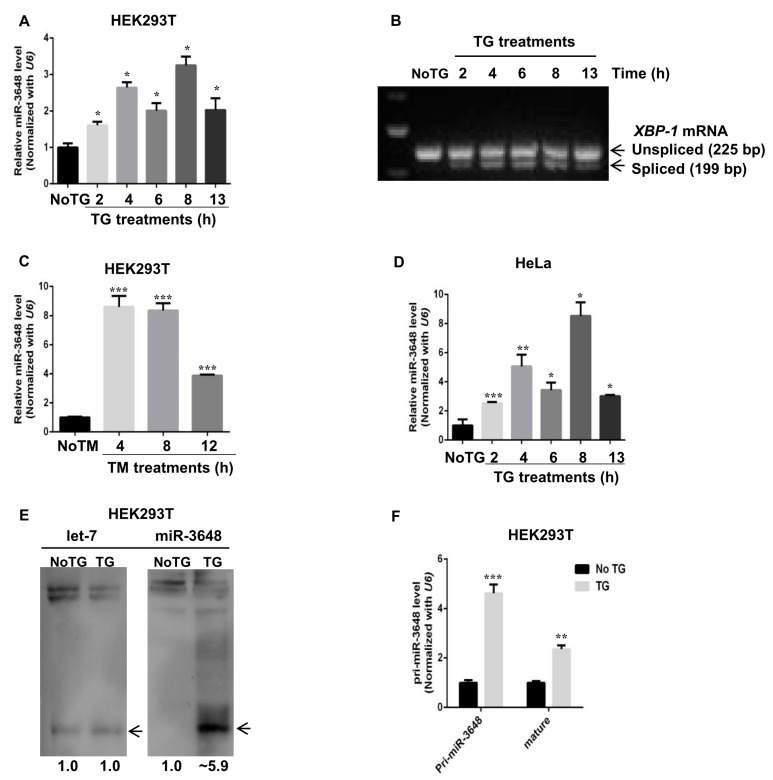
miR-3648 was upregulated under ER stress: (**A**) qPCR analysis of mature miR-3648 levels in HEK293T cells after TG treatment (300 nM) for indicated time points; (**B**) the cytoplasmic splicing of XBP-1 mRNA in response to TG treatment at different time points was detected by separating the RT-PCR product in an agarose gel; (**C**) qPCR analyses of miR-3648 expression levels in HEK293T cells after TM treatment (300 nM) for indicated time points; (**D**) qPCR analysis of miR-3648 expression levels in HeLa cells after TG treatment (300 nM) for indicated time points; and (**E**) Northern blot of miR-3648 and *let-7*. *let-7* was used as loading control. HEK293T cells were either untreated or treated with TG for 8 h. Bands were quantified relative to *let-7* with Image J (Ver 1.51j8, NIH, Bethesda, MD, USA, available online: https://imagej.nih.gov/ij). Arrowheads indicates mature miRNA bands. (**F**) qRT-PCR analyses of primary and mature forms of miR-3648 in untreated or TG treated HEK293T cells. * *p* < 0.05; ** *p* < 0.01; *** *p* < 0.001. *p* values were determined with two-tailed student’s *t* test. All data were from three repeats. Error bars represent standard deviation S.D.

In an effort to identify microRNAs upregulated upon ER stress, we noticed that levels of mature miR-3648 were increased when cells were treated with TG, a drug commonly used to induce ER stress, in HEK293T cells (Figure 2A). UPR induction upon TG treatment was confirmed by the cytoplasmic splicing of *XBP-1* [33] (Figure 2B). Similarly miR-3648 was induced when HEK293T cells were treated with Tunicamycin (TM), another drug that can induce ER stress (Figure 2C). We also observed this induction of miR-3648 in HeLa cells treated with TG (Figure 2D). We next examined miR-3648 levels with Northern blots, and mature miR-3648 was significantly increased with TG treatment for 8 h (Figure 2E). However, as a comparison, no change was observed for the level of *let-7* (Figure 2E), an abundant miRNA that regulates cellular differentiation in the developing organism [34].

To know at which stage the induction of miR-3648 happened, we examined levels of pri-miR-3648 [35] (Figure 2F). Levels of pri-miR-3648 and mature miR-3648 were significantly increased with TG treatment (Figure 2F). These results demonstrated that levels of mature miR-3648 increased in cells under ER stress, and it was highly possible due to the transcriptional activation of pri-miR-3648.

### 2.3. miR-3648 Directly Targeted the 3′ UTR of APC2

**Figure 3:**
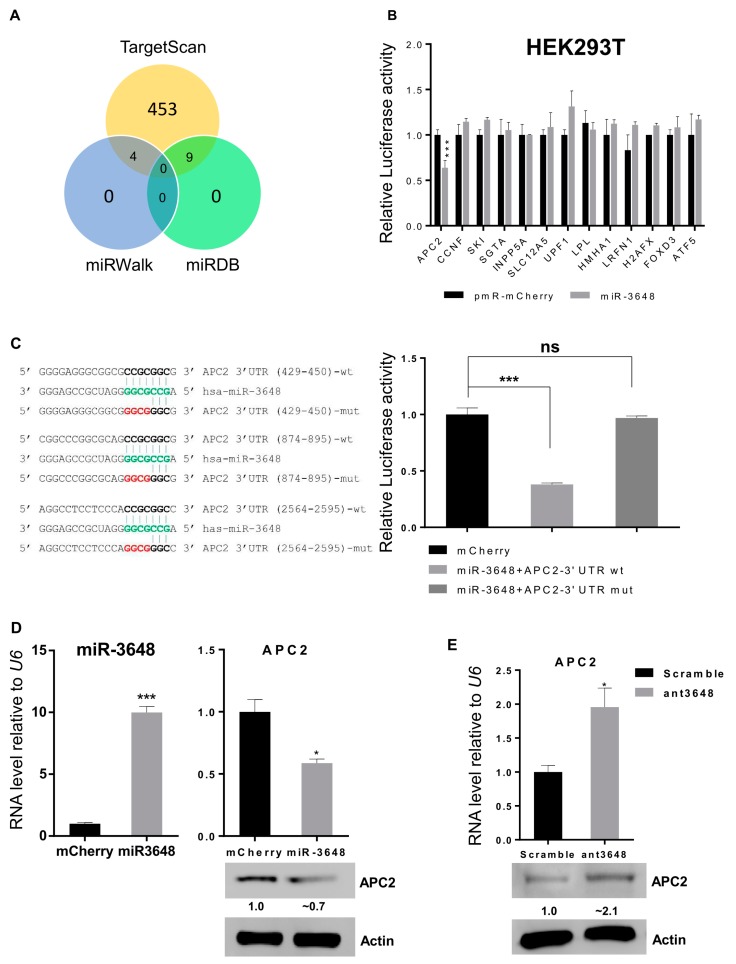
miR-3648 targeted the APC2 3′ UTR: (**A**) Venn diagram shows the predicted targets of miR-3648; (**B**) HEK293T cells were co-transfected with miR-3648 or pmR-mCherry (mCherry) with pRL-null (Renilla plasmid) and firefly luciferase reporter plasmids harboring the corresponding 3′ UTR. The ratio of the reporter (*Firefly*) to control (*Renilla*) in relative luminescence units was plotted; (**C**) The 3 binding sites of miR-3648 in 3′ UTR of the APC2 mRNA are shown in bold black. The nucleotides mutated in 3′ UTR of APC2 are highlighted in red. Nucleotides in green color represents miR-3648 seed region. The overexpressed miR-3648 had no effect on the mutated APC2 3′ UTR when examined with luciferase assays; (**D**) miR-3648 was overexpressed in HEK293T cells, and the relative expression levels of APC2 were examined by qRT-PCR (for the mRNA) and Western blots (for the protein); (**E**) levels of APC2 were examined by qRT-PCR (for the mRNA) and Western blots (for the protein) in HEK293T cells treated with the scrambled control or antagomir (ant3648). * *p* < 0.05; *** *p*< 0.001; ns, not significant. *p* values were determined with two-tailed student’s *t* test. All data were from triplicates. Error bars represent S.D.

In order to identify potential targets of miR-3648, we used three algorithms i.e. Targetscan, miRDB and miRWalk, and 13 target genes in common were identified [36,37,38] (Figure 3A). We then performed luciferase reporter assays for 3′ UTR of all these predicted targets. The relative luciferase activity of reporter with APC2 3′ UTR was significantly repressed by miR-3648, while no effect was observed on the luciferase activity for all the other 3′ UTR reporters (Figure 3B). Further, we mutated all the three predicted binding sites of miR-3648 within the 3′ UTR of APC2, and the suppressive effect of miR-3648 was then abolished (Figure 3C). When miR-3648 was overexpressed, both the mRNA and protein levels of APC2 were downregulated (Figure 3D). Conversely, when the cells were transfected with miR-3648 antagomir (ant3648), both the mRNA and protein levels of APC2 were upregulated (Figure 3E). These results showed that APC2 was the only miR-3648 target among the 13 predicted genes, and it was a direct target with miR-3648 binding sites in its 3′ UTR.

### 2.4. APC2 Was Regulated by miR-3648 under ER Stress

**Figure 4:**
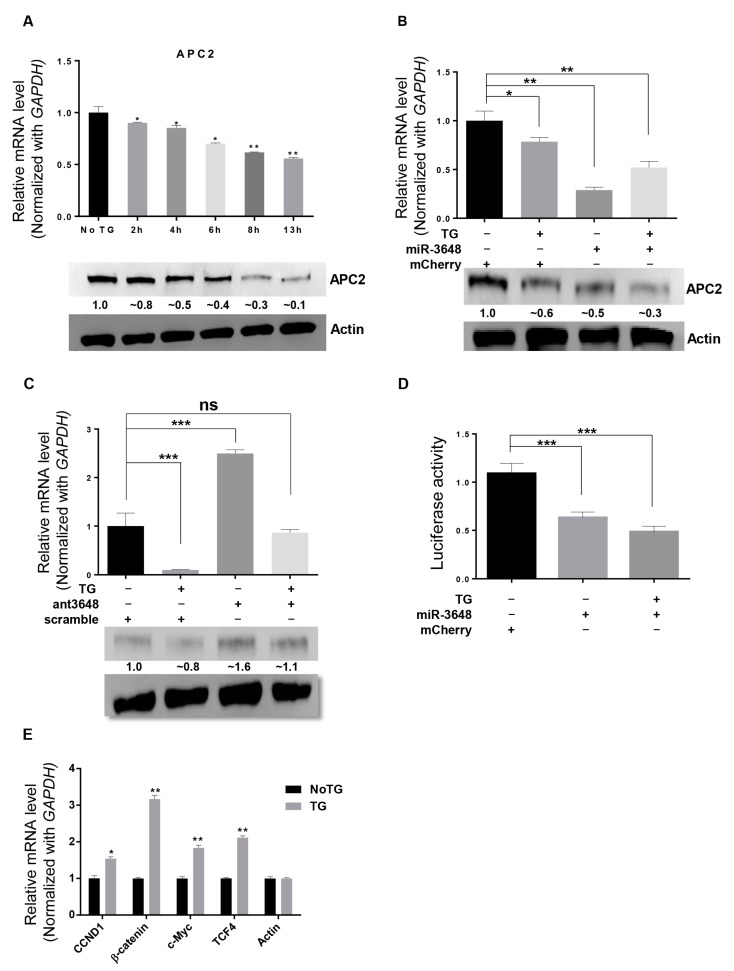
APC2 was regulated by miR-3648 under ER stress: (**A**) APC2 mRNA levels (qRT-PCR) and protein levels (Western blots) in HEK293T cells after TG treatment at different time intervals; (**B**) APC2 mRNA levels (qRT-PCR) and protein levels (Western blots) in HEK293T cells with or without TG treatment and miR-3648 overexpression. Although the levels of APC2 mRNA seems to increase when miR-3648 was overexpressed in stressed cells, the change was statistically not significant (*p* = 0.09). Protein bands were quantified with Image J; (**C**) APC2 mRNA levels (qRT-PCR) and protein levels (Western blots) in HEK293T cells with or without TG treatment and miR-3648 antagomir (ant3648). Protein bands were quantified with Image J; (**D**) APC2 3′ UTR Luciferase assays in HEK293T cells with or without TG treatment and miR-3648 overexpression. (**E**) qRT-PCR analyses of Wnt/β-catenin pathway genes in ER stressed HEK293T cells. * *p* < 0.05; ** *p* < 0.01; *** *p* < 0.001. *p* values were determined with two-tailed student’s *t* test. All data were from three repeats. Errors bars represent S.D.

We next examined whether TG treatment could affect APC2 levels. Decreased APC2 mRNA and protein levels were found through the time course of ER stress (Figure 4A). To investigate whether these decreases of APC2 levels in ER stressed cells were due to increases in miR-3648 levels (Figure 2 and Figure 3), we performed experiments to overexpress or block (with antagomir) miR-3648 in cells under ER stress (Figure 4B,C). Both the APC2 mRNA and protein levels were further downregulated when miR-3648 was overexpressed in ER stressed cells (Figure 4B). Conversely, miR-3648 antagomir significantly increased the APC2 mRNA and protein levels in ER stressed cells (Figure 4C). Luciferase assays confirmed that miR-3648 could regulate APC2 by targeting the 3′ UTR of APC2 in ER stressed cells (Figure 4D). These results revealed that elevated levels of miR-3648 suppressed the expression of APC2 in cells under ER stress.

APC2 is an interacting partner of β-catenin, and negatively regulates β-catenin signaling pathway [25,27]. We sought to find out whether decreased APC2 levels could activate the downstream Wnt/β-catenin pathway genes in ER stressed cells. Indeed, the expression levels of target genes of Wnt pathway such as *CCND1*, *β-catenin*, *c-Myc*, and *TCF4* were upregulated in stressed cells (Figure 4E).

### 2.5. Upregulation of miR-3648 Increased Cell Proliferation

**Figure 5:**
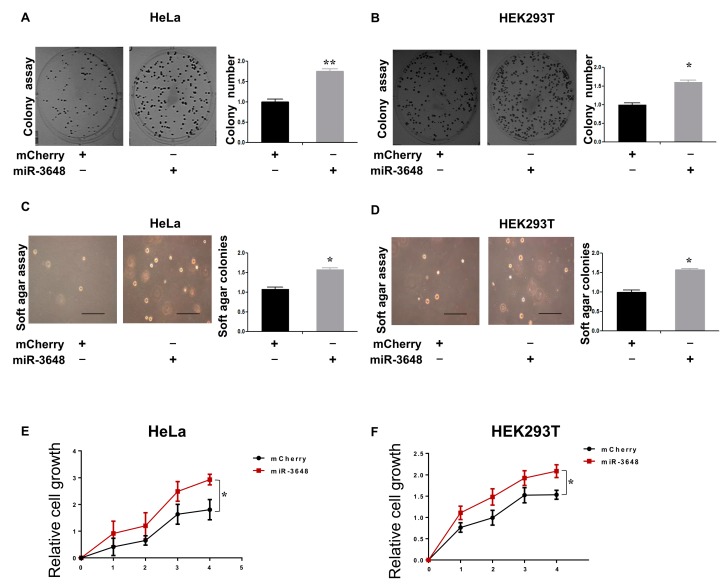
miR-3648 upregulation increased cell proliferation in both HeLa and HEK293T cells: (**A**,**B**) representative micrographs and quantification of colony formation assays in cells overexpressing miR-3648; (**C**,**D**) representative micrographs and quantification of soft agar assays in cells overexpressing miR-3648; scale bar represents 100 µm (**E**,**F**) quantification of cell proliferation (MTT assays) for cells overexpressing miR-3648. * *p* < 0.05; ** *p* < 0.01; *p* values were determined with two-tailed student’s *t* test. All data were from three repeats. Error bars represent S.D.

APC2 is a well-known tumor suppressor, mainly due to its suppressive role in the Wnt signaling [27,28,30]. Thus far, our findings suggested that miR-3648 was induced, and then APC2 was suppressed, in ER stressed cells. Therefore, miR-3648 might play roles in tumorigenesis, especially in the context of ER stress. To examine this, we overexpressed miR-3648 in HeLa and HEK293T cells (Figure 5). We observed cells overexpressed with miR-3648 formed significantly more colonies in cells compared to control group (Figure 5A,B). Overexpression of miR-3648 could also enhance the anchorage-independent growth (examined with soft agar assays) as compared to control group (Figure 5C,D). Cell proliferation was similarly increased in cells with miR-3648 overexpression (Figure 5E,F).

### 2.6. Suppression of miR-3648 Decreased Cell Proliferation

**Figure 6:**
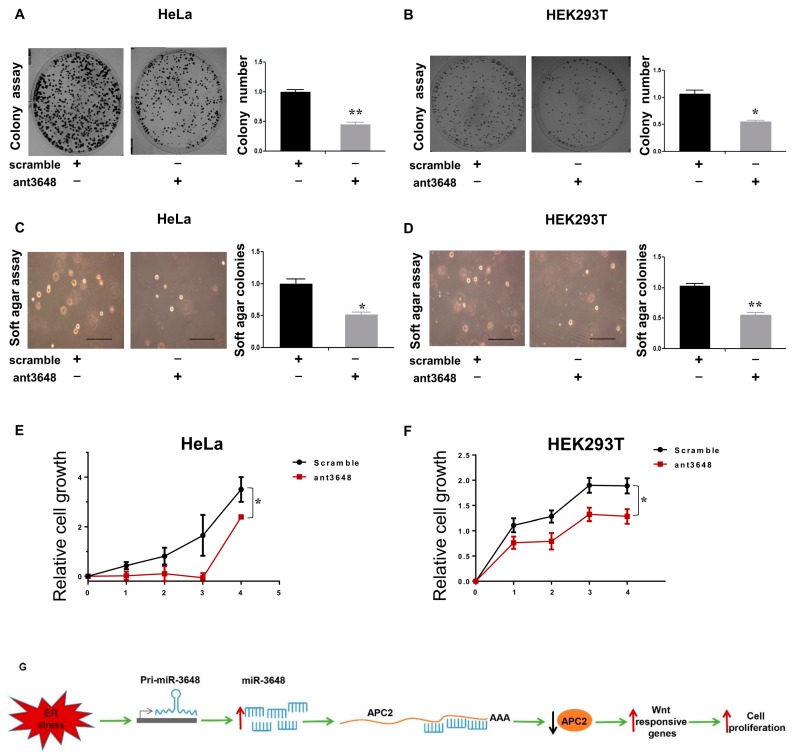
Suppression of miR-3648 decreased cell proliferation: (**A**,**B**) representative micrographs and quantification of colony formation assays after blocking miR-3648; (**C**,**D**) representative micrographs and quantification of soft agar assays after blocking miR-3648; scale bar represents 100 µm (**E**,**F**) quantification of cell proliferation (MTT assays) after blocking miR-3648. * *p* < 0.05; ** *p* < 0.01; *p* values were determined with two-tailed student’s *t* test. All data were from three repeats. Error bars represent S.D; (**G**) proposed model: miR-3648 was induced at the transcriptional level by ER stress, and then miR-3648 suppressed the expression of APC2, a negative regulator of Wnt signaling pathway. These regulations contributed to the stimulated cell proliferation in ER stressed cells.

We then suppressed the function of miR-3648 by applying the miR-3648 specific antagomir (ant3648) (Figure 6). Cells treated with ant3648 decreased the colony formation as compared to scramble treated cells (Figure 6A,B). Similarly, cells treated with ant3648 repressed the anchorage independent cell growth (Figure 6C,D). Cell proliferation was also decreased in cells with ant3648 treatment (Figure 6E,F).

## 3. Discussion

Our study showed that the human specific miR-3648 was induced at the transcriptional level by ER stress. The increased levels of miR-3648 then suppressed the expression of APC2, a negative regulator of cell proliferation and Wnt signaling pathway. All these regulations would eventually contribute to stimulated cell proliferation under ER stress (Figure 6G).

We demonstrate that expression level of pri-miR-3648 along with mature miR-3648 is up-regulated during ER stress (Figure 2F). This indicates the possibility that the induction of this miRNA is regulated at transcriptional level. Further study is required to explore the mechanism of transcriptional regulation of miR-3648 expression during ER stress.

miR-3648 is also shown to be up-regulated in multiple high throughput researches in conditions such as Japanese Encephalitis Virus infection in microglia cells, HBV-positive HCC cells, and also in Renal cell carcinoma (RCC) and Upper tract carcinoma (UT-UC) [39,40,41]. These studies along with our study indicate that miR-3648 may be a tumor promoting miRNA, and is upregulated in multiple conditions that trigger ER stress, as ER stress can also be induced during viral infections [42].

APC2 is identified as the target of miR-3648 in ER stressed cells. APC2’s role is mainly as a negative regulator of Wnt/β-catenin pathway [30]. Wnt signaling is generally upregulated in ER stress mainly via the so-called CHOP-Wnt pathway [15,43]. In this study, we also find increased expression level of target genes of Wnt/β-catenin signaling pathway (Figure 4E), and our data regarding miR-3648 and APC2 add a novel link between ER stress and Wnt/β catenin signaling in human cells. Wnt/β-catenin pathway plays important roles in cellular differentiation and proliferation, and abnormal activation of this pathway may lead to various human cancers [44,45,46].

Many human specific miRNAs such as miR-3648 have been identified, however, most of them have no identified targets or functionality yet [32,47]. It is interesting to notice that among the 13 predicted targets of miR-3648, only APC2 is the direct target of miR-3648 (Figure 3). It would be tempting to propose that newly evolved microRNAs may have less number of targets as compared to more ancient miRNAs. miR-3648 is expressed in essentially all cells and tissues examined (Figure 1), is induced, and plays critical roles in ER stressed cells. Therefore, this human specific microRNA may be a newly added molecule in the cellular responses to stresses. Whether APC2 in other mammals is suppressed in ER stress by some other microRNAs or even via alternative pathways remains an interesting topic of investigation. Roles of miR-3648 in ER stress and other forms of cellular stress in human cells also deserve further study.

## 4. Materials and Methods

### 4.1. Construction of Plasmids

For the functional analysis of miR-3648, partial segments of the mRNA 3′ UTR containing the miR-3648 binding sequences of *APC2*, *CCNF*, *SKI*, *SGTA*, *INPP51*, *SLC12A5*, *UPF1*, *LPL*, *HMHA1*, *LRFN1*, *H2AFX*, *FOXD3* and *ATF5* were PCR amplified from cDNA prepared from RNA of HEK293T cells. The PCR product was then sub cloned into the *XbaI* site downstream of the stop codon in the pGL3-control firefly luciferase reporter vector. miRNA expression plasmid was constructed by inserting DNA fragment containing pre-miRNA coding sequence between the *HindIII* and *BamHI* sites of pmR-mCherry (Clontech, Mountain View, CA, USA). The correct orientation of 3′ UTR fragments and pre miRNA coding sequences in plasmid DNA constructs was confirmed by sequencing. All the primers used for plasmid construction are given in Table S1.

### 4.2. Cell Culture, Transfections, TG and TM Treatments

HeLa and HEK293T cells were cultured under standard conditions with DMEM (GE Healthcare, South Logan, UT, USA) plus 10% heat inactivated fetal bovine serum (FBS) at 37% and 5% CO_2_. Plasmid transfection was performed with Lipofectamine2000 (Invitrogen, Carlsbad, CA, USA) according to the supplier’s protocol. 2′-*O*-methyl RNA/DNA antisense oligonucleotides (ASOs) which were modified by changing the five nucleotides at 5′ and 3′ end into 2′-*O*-methylribonucleotides, were synthesized by RiboBio (Guangzhou, China). The antagomir transfections were performed at a concentration of 200nM with Lipofectamine2000 (Invitrogen) according to the supplier’s protocol. For Thapsigargin (TG) and Tunicamycin (TM) treatments, actively growing cells were incubated for the indicated times with a 300 nM TG or TM.

### 4.3. PCR Reactions

Total RNA was isolated using Trizol reagent (Invitrogen) according to the manufacturer’s instructions. DNA was eliminated with nuclease-free DNase (Promega, Madison, WI, USA). For RT–PCR, complementary DNA was synthesized from RNA with a GoScript Reverse Transcription System (Promega) according to the manufacturer’s protocol, with the corresponding primers. About 500 ng RNA was used for cDNA preparation. Quantitative real-time PCR was performed with GoTaq SYBR Green qPCR Master Mix (Promega) on a PikoReal 96 real-time PCR system (Thermo Scientific, Waltham, MA, USA) according to standard procedures. All PCR products were sequenced for confirmation. All the primers are given in Table S1.

### 4.4. miRNA Isolation and Northern Blot

Sense and antisense digoxigenin-labeled DNA probes were purchased from Generay Biotechnology. Fifteen micrograms of miRNA and the Riboruler, low range RNA ladder (Thermo scientific), were mixed with 2× RNA loading dye (Thermo scientific). These mixtures were then denatured at 60 °C for 5 min followed by fractionated on 15% Urea PAGE Gel (Criterion, BioRad, Hercules, CA, USA) for 1 h at 120 Volts. RNA was then transferred onto Hybond-N^+^ membranes (GE Healthcare) by electro transfer method at 200 mA for 4 h on ice. The membrane was baked for 1 h at 80 °C followed by UV cross linking for 2 min. The membrane was pre-hybridized for 30 min at 42 °C with pre-warmed (42 °C) Dig easy Hub granules. The probe was denatured at 100 °C for 5 min followed by rapid cooling on ice for 5 min. This probe was then added to fresh Dig easy Hub granules. Hybridization with probes was performed at 42 °C overnight. Detection was performed according to the manufacturer’s protocol (Roche, DIG Northern Starter Kit, Basel, Switzerland). Images were taken with an ImageQuant LAS4000 Biomolecular Imager (GE Healthcare). The probe sequences are given in Table S1.

### 4.5. Luciferase Assay

HEK293T cells (1 × 10^6^) were transfected with 1μg of pmR-mCherry-miR-3648 or empty vector control, 1 μg of pGL3 Control Vector carrying the corresponding 3′ UTR and 10 ng pRL-TK renilla plasmid. In the case of TG experiments, 8 h before cells lysis, 300 nM of TG was added with fresh media. Luciferase assays were performed 48 h after transfection using Dual Luciferase Reporter Assay System (Promega) according to the manufacturer’s protocol. Briefly, cells were lysed with passive lysis buffer at room temperature for 20 min. The luciferase assay buffer II was then added, and firefly luciferase (F-luc) activity was immediately read using a Fluoroskan Ascent FL microplate reader (Thermo Scientific). Next, Stop & Glo Buffers with Stop & Glo substrates were added and mixed briefly. Renilla luciferase (R-luc) activity was immediately read. F-luc activity was normalized to R-luc activity to account for variation in transfection efficiency.

### 4.6. Western Blots

Western blots were performed from the cells transfected with miR-3648 overexpressing plasmids or ant-3648 after 48 h. While Western blots for TG treatment cells were performed at the indicated times post treatments, wor Western blots, samples were separated on 10% SDS–PAGE gels and then transferred to PVDF membranes (Millipore, Billerica, MA, USA). Proteins on the membranes were blocked with 5% non-fat milk for 1 h at room temperature. The membranes were then incubated with primary antibodies in 5% non-fat milk over night at 4 °C. After washing the membranes three times with TBST, these were incubated with the respective secondary antibodies. Membranes were processed according to the ECL Western blotting protocol (GE Healthcare). The following primary antibodies were used in Western blots: anti-APC2 (Abcam, cat#113370) and anti-Actin (Signalway antibody, 21338).

### 4.7. Colonogenic Assay

HeLa or HEK293T cells in 6-well plates were transfected with the corresponding plasmids or oligos, with or without TG treatment. After 48 h, cells were trypsinised and 500 cells were plated in triplicate into each well of 6-well plate. The plates were incubated for 14 days at 37 °C, in a 5% CO_2_. The media was changed after every 3 days. Finally the cells were washed twice with PBS and colonies were fixed with 37% (*v*/*v*) formaldehyde for 10 min at room temperature. The colonies were stained with 0.1% crystal violet dye for 20 min. The wells were rinsed with water carefully. The colonies were counted for quantification. 

### 4.8. Soft Agar Assay

Soft agar plates were prepared using six-well culture plates. Briefly, 3 mL 0.5% Noble agar containing DMEM medium with 10% FBS were poured into each well to form a base. HeLa or HEK293T cells in 6-well plates were transfected with the corresponding plasmids or oligos, with or without TG treatment. After 48 h, cells were trypsinised and a total of 2000 cells were counted and were diluted in 1 mL DMEM medium and then further diluted in 0.7% Noble agar to give a final concentration of 0.35% noble agar. This mixture of cells was poured on the top of hardened agar base and allowed to solidify. One milliliter of DMEM medium was added to the cells after 10 min of incubation at room temperature. The dishes were incubated at 37 °C in a 5% CO_2_. The media was changed after every 3 days. The colonies were counted after 14 days. 

### 4.9. Cell Proliferation Assay

Cell viability was measured with the MTT Cell Proliferation and Cytotoxicity Detection Kit (Keygentec, Nanjing, China) according to the manufacturer’s recommendations. Cells in 96-well plates were plated at a density of 2 × 10^3^ per well. MTT reagents were added at indicated time points. Four hlater the supernatant was removed, and DMSO was added to dissolve the blue precipitates. The amount of live cells was determined by the OD value, which was measured by a plate reader (MultiSkan Go, Thermo Scientific).

## 5. Conclusions

We found that miR-3648, a human specific microRNA, was upregulated upon ER stress. Adenomatous polyposis coli 2 (APC2) was found to be the direct target of miR-3648. APC2 is a tumor suppressor and a negative regulator of Wnt signaling. We demonstrated that levels of APC2 were downregulated when miR-3648 was induced. Inhibition of miR-3648 increased APC2 levels and decreased the cell proliferation. Conversely, when miR-3648 was overexpressed, APC2 levels were decreased and the cell growth increased. Our findings demonstrated that ER stress mediated induction of miR-3648, which then downregulated APC2 and increase cell proliferation. Since this miRNA is also found to be upregulated in several other diseases such as HCC, RCC and UT-UC, the downregulation of APC2 by miR-3648 might be associated with these diseases.

## Supplementary Materials

Supplementary materials can be found at www.mdpi.com/1422-0067/18/7/1375/s1.

## Abbreviations

APC2	Adenomatous polyposis coli 2
ER	Endoplasmic Reticulum
MTT	3-(4,5-dimethylthiazol-2-yl)-2,5-diphenyltetrazolium bromide
TG	Thapsigargin
TM	Tunicamycin
