# Supplementary material for: Induction of miR-3648 Upon ER Stress and Its Regulatory Role in Cell Proliferation
Source: Int J Mol Sci. 2017 Jun 29;18(7):1375. doi: 10.3390/ijms18071375 (PMC5535868; doi:10.3390/ijms18071375)
Supplement: Supplementary file 1 [file ijms-18-01375-s001.pdf]

Induction of miR-3648 upon ER stress and its Regulatory Role in Cell Proliferation

Farooq Rashid, Hassaan Mehboob Awan, Abdullah Shah, Liang Chen and Ge Shan *

Table S1. Oligos used in this study

Oligo name	Oligo sequence	Purpose
APC2-F	AGATCGCCGTGTAATTCTAGACTTCCCCTGTCGGAAGCCGTTG	Plasmid for 3′ UTR luciferase reporter assays
APC2-R	GCCGGCCGCCCCGACTCTAGAGACGGTGTCGTCCTTACG	
CCNF-F	AGATCGCCGTGTAATTCTAGAGTGTGTCAGCACATTTGCCG	Plasmid for 3′ UTR luciferase reporter assays
CCNF-R	GCCGGCCGCCCCGACTCTAGACATCTGCCACTATCCTCC	
SKI-F	AGATCGCCGTGTAATTCTAGAGTCCTCTGCTTGCTGGAAC	Plasmid for 3′ UTR luciferase reporter assays
SKI-R	GCCGGCCGCCCCGACTCTAGACCTCAGTAAACCAAGGGC	
SGTA-F	AGATCGCCGTGTAATTCTAGAACCCGAAGGAAGCCTTCTGG	Plasmid for 3′ UTR luciferase reporter assays
SGTA-R	GCCGGCCGCCCCGACTCTAGATTCCCCACACGTCACGCC	
INPP5A-F	AGATCGCCGTGTAATTCTAGAGGAACTTTCCCAGCGGATC	Plasmid for 3′ UTR luciferase reporter assays
INPP5A-R	GCCGGCCGCCCCGACTCTAGAGCTTAGGAACCAGACCGG	
SLC12A5-F	AGATCGCCGTGTAATTCTAGAGAACCAGGACCTGCCACCC	Plasmid for 3′ UTR luciferase reporter assays
SLC12A5-F	GCCGGCCGCCCCGACTCTAGAGCCATAAAGCATTAAATGGG	
UPF1-F	AGATCGCCGTGTAATTCTAGAGGTGGCGGCGGAAGAGCT	Plasmid for 3′ UTR luciferase reporter assays
UPF1-R	GCCGGCCGCCCCGACTCTAGAGCCTTGGTACAGTGCGCC	
LPL-F	AGATCGCCGTGTAATTCTAGACTGGGCGAATCTACAGAAC	Plasmid for 3′ UTR luciferase reporter assays
LPL-R	GCCGGCCGCCCCGACTCTAGAGAGTGAGTTGGCTCTGTG	
HMHA1-F	AGATCGCCGTGTAATTCTAGAGCTGGGGTGGGGCTGGGA	Plasmid for 3′ UTR luciferase reporter assays
HMHA1-R	GCCGGCCGCCCCGACTCTAGACGTGCCTGTGAAAGAACC	
LRFN1-F	AGATCGCCGTGTAATTCTAGATGGGTGCCGCAGACCAAAC	Plasmid for 3′ UTR luciferase reporter assays
LRFN1-R	GCCGGCCGCCCCGACTCTAGATTCTGCTGCGGCGGAGGGT	

H2AFX-F	AGATCGCCGTGTAATTCTAGAAGGCCTCCCCATGCCACCA	Plasmid for 3′ UTR luciferase reporter assays
H2AFX-R	GCCGGCCGCCCCGACTCTAGACCCCAATGCCTAAGGTTC	
FOXD3-F	AGATCGCCGTGTAATTCTAGAACGCGCCAATGGCCGGGA	Plasmid for 3′ UTR luciferase reporter assays
FOXD3-R	GCCGGCCGCCCCGACTCTAGAGTTGTGTACAACACGATGG	
ATF5-F	AGATCGCCGTGTAATTCTAGAGGGCAGGGGTGTGGCTTCTG	Plasmid for 3′ UTR luciferase reporter assays
ATF5-R	GCCGGCCGCCCCGACTCTAGAGCCAGAGGAAATGTTTCG	
miR-3648-F	GGTACCGCGGGCCCGGGATCCGTGGGCTTCCCGGAGGGTT	Plasmid for 3′ UTR luciferase reporter assays
miR-3648-R	AGTTATCTAGATCCGGTGGATCCAAGAGCGGGCCGGGAGAAG	
CCND1-F	ATCAAGTGTGACCCGGACTG	qRT-PCR
CCND1-R	CCTTGGGGTCCATGTTCTGC	
β-catenin-F	ACGAGCTGCTATGTTCCCTG	qRT-PCR
β-catenin-R	GATGGTTCAGCCAAACGCTG	
c-Myc-F	CCCTCCACTCGGAAGGACTA	qRT-PCR
c-Myc-R	GCTGGTGCATTTTCGGTTGT	
TCF4-F	CGACTTCCCCTGACCTGAAC	qRT-PCR
TCF4-R	CTCATCACCCTCGTCATCGG	
Actin-F	TGACAGGATCGAGAAGGAGA	qRT-PCR
Actin-R	CGCTCAGGAGGAGCAATG	
miR-3648-F	CACGCAGCCGCGGGGAT	qRT-PCR
miR-3648-R	CCAGTGCAGGGTCCGAGGTA	
Pri-miR-3648-F	GTGGTCTCTCGTCTTCTC	qRT-PCR
Pri-miR-3648-R	ACGGACGCCTCGGGGAAG	
Scramble	A*A*A*G*U***ATCTGTAAAAG**U*G*A*A*C*	Inhibitor for miR-3648
ant3648	C*C*C*U*C***GGCGATCCCCG**C*G*G*C*U*<br>**Bold** letters represent DNA sequence<br>* represents 2′O methylation	
